# Alleged Poisoning by Morphine—Superior Court of Buffalo—Frances C. Dustin, Administratrix of James E. Dustin, Deceased, against Merrell Eugene Shaw, M. D.

**Published:** 1867-11

**Authors:** 


BUFFALO

Medical and Surgical Journal.

VOL. VII. NOVEMBER, 1867. No. 4.

### Original Communications.

ART. I.—*Alleged Poisoning by Morphine—Superior Court of Buffalo—Frances C. Dustin, Administratrix of James E. Dustin, deceased, against Merrell Eugene Shaw, M. D.*

This action was brought on before Hon. George W. Clinton and a jury, November 12, 1867, under a statute of this State, enacted in 1847, permitting an executor or administrator to maintain an action at law against a physician or surgeon in the case where the death of the testator or intestate is occasioned by mal-practice, in a similar manner to that in which an action for mal-practice was previously allowed to the injured person himself when the injury did not result in death.

The complaint in this cause alleged that about two o'clock in the morning on the 30th day of June, 1867, James E. Dustin, the plaintiff's intestate, being "seized by a sudden and painful attack in the right iliac region," employed the defendant in his capacity of a physician; and that the defendant then administered to him "subcutaneous injections over the right iliac region of large quantities of a poisonous drug called morphine, which entered into the system of said deceased, and that thereby and in consequence thereof at about the hour of nine and a half o'clock in the morning of said 30th day of June the said Dustin died;" and that the death of said Dustin was so caused by the unskillfulness, want of proper care, neglect, wrongful act and default of the defendant. The plaintiff claimed to recover the sum of $5,000.

The answer of the defendant admitted that at the time mentioned he had given morphine to the deceased by subcutaneous injection, but he denied that he had given it in an excessive dose, or improperly, or that the death of Dustin was caused by morphine, or that the defendant had been guilty of any unskillfulness, negligence, want of care, etc., alleging on the contrary that the treatment was in all respects judicious and proper.

The cause being called and a jury empanneled the plaintiff's counsel read in evidence the deposition of the defendant taken at a coroner's inquest on the body of the deceased on the 1st day of July, in which Merrell E. Shaw being sworn, said: I am a practicing physician and surgeon, No. 209 Seneca street; I knew the deceased; I was called about one and a half o'clock on Sunday morning by Mrs. Dustin; she said her husband was ill, and wanted my attendance; I accordingly went with her to her house; he complained of pain in right iliac region; he said he had an attack about the fourth of July, 1866; he had pain and difficulty in making water, and was attended by Dr. Mead; I treated him for colic pains; he told me he had eaten some greens and drank some beer; I gave a subcutaneous injection of morphine, half a grain as near as I can judge, as I have been in the habit of giving it before; I call that a medium dose; I asked him if he felt relieved; he said he was not relieved; subcutaneously, anodynes have almost immediate effect in relieving pain; I have frequently given them without producing any stupor or deleterious effect; Mrs. Dustin went after mustard; I think I repeated the dose during her absence, about the same quantity at an interval of half an hour after the first; Mrs. Dustin asked him if he felt any easier; he said he did; I then went to my office; before going I left six powders; if the pain was not relieved to give one of the powders in half an hour; they were anodyne powders of morphine, one-half grain; they said they had not given any of them; I was called there about five o'clock in the morning; I saw the patient; he was comatose or stupid, breathing stertorously; the pupils of the eye rather contracted; his pulse rather irregular, varying from 90 to 112; I could see froth by opening his mouth; I tried to get him up on his feet;

I went to the druggists, Smith & Hickey, for some ammonia; I did all I could to stimulate and resuscitate him; I gave him some cayenne pepper and whisky; he let some down; it was rather an involuntary movement, the muscles of the throat contracted; I went up after my father, who came down with me; the dose I gave is about what is given; if he died from the effects of the morphine, it must have been from the idiosyncrasy of the patient; I think he died from the congestion of the brain; from the morphine or stimulants taken into the system; he died at about nine and a half o'clock on Sunday morning.

Frances E. Dustin was called and sworn on the part of the plaintiff, and testified: Am the widow and administratrix of the deceased; he died at half past nine o'clock, June 30th; he was a railroad engineer on the Erie road; I have three children by him; I saw him about seven o'clock on Saturday evening, June 29th; he appeared to be well; he came in and and ate his supper; I sat with him; then he went into the dining-room and laid down and went to sleep; he slept till about eleven o'clock; I went to him and asked if he was going to sleep on the lounge all night; he said he would go to bed in a few minutes; I went to bed, and to sleep; I did not hear him get in bed; at about half past one he got out of bed; he asked me if we had any mustard in the house; I said no, I would get some; I asked him if he was sick; he said, no more than having a pain in his stomach, a pain across the bowels; I woke up a neighbor and got some mustard; I put it on where pain was on his bowels; he said it didn't ease him; I then went for young Dr. Shaw; he came with me to the house; Dustin laid down on the lounge when we came in; the Doctor felt his pulse; I asked if I should get some more mustard; he said, yes; my sister went with me for it to the drug store in Seneca street; got half a pound; this was about two o'clock Sunday morning; when I came in he said his pain was easier, but he felt sick at his stomach and dizzy, as if he were drunk; he was after being sick at his stomach and vomiting; I got the mustard ready and put it on where he complained of the pain; the Doctor was still there; he got up to go away; I asked if he thought he would get along; he said he thought so; he said he would leave six powders, and I should give one every half hour if the pain came back, but not to give him any unless the pain came on pretty freely; told me to come in the morning and let him know how Dustin was; Dustin asked for the slop-pail, said he was sick at his stomach; I brought it; he was on the lounge; he felt easier, but was dizzy and sick at his stomach; he said the Doctor gave him the medicine with a small syringe through the skin; I sat down near him, was sleepy; he told me to go to bed; he said he should go himself in a few minutes; this was about three o'clock; I laid down in the front bed-room and went to sleep; didn't wake up till about half past four; the baby woke me up; heard a dreadful snoring; I called him by name; told him to wake up; I came into the dining-room; he lay on his back just as I left him; I saw the froth and blood was running out of his mouth; tried to get a drink of cold water down; I thought he was in a fit; the tongue was set right up in the roof of his mouth; I unbuttoned his shirt and rubbed him; my sister got up; I called Mr. Matthews' people; Matthews and Chapman came in; Chapman went for the Doctor; Dr. Shaw came and said he was in a heavy sleep; he began rubbing him; told me to get warm water and some cloths; asked for liquor and cayenne pepper; he put a little in his mouth; I didn't see him swallow it; he didn't move, or open his eyes or speak; they tried to set him on his feet; he couldn't stand; the Doctor went for his father and to the drug store; came back with his father; the young Doctor rubbed something on him.

Henry Beathig called and sworn for the plaintiff, testified:— I am a homœopathist; I commenced this year; have practiced before, but not made a business of it; I passed an examination before the homœopathic board of Erie county; I was called to Carroll street on Sunday, the last day of June; I found some persons there; I did not examine the patient; he was in a comatose condition; I was three or four paces from him, room crowded; I declined to do anything as doctors were there; I gave belladonna, 30th attenuation, an antidote to morphine; the pills are pure sugar, moistened with that attenuation; I gave them to young Hager; don't knew whether he administered them; such breathing could be produced by an overdose of morphine; there is a great difference whether morphine is injected under the skin or given to the patient by the stomach; it would take less to kill a man if injected; I think a grain would kill.

Cross-examined.—I was educated at Breslau, Germany; from 1832 to 1834 was a minister; ran away in 1850; commenced to practice for a living this present year; when I first came to this country I had a soap factory; was then a school teacher for about a year; then a daguerrian for about five, six or seven years; then went into a drug store; ran it for five years; got my document from the Erie County Homœopathic Medical Society; have no diploma from any medical college; drug store was on corner of Main and Genesee streets; other causes than morphine would produce breathing such as Dustin exhibited; any man in a comatose state would breathe in the same way; I never injected morphine; never saw it done.

Frances E. Dustin re-called.—I remember the box of pills; it was left on the mantel-piece; *the baby ate the pills*, and they did not hurt him.

The plaintiff here rested.

Julius F. Miner called and sworn on the part of the defendant, testified: I have practiced medicine and surgery twenty years; I knew Dr. Shaw, the defendant in this case; I saw James E. Dustin previous to his death; found him early in the morning, breathing stertorously, insensible, pulse at times almost indistinct; the pupils of the eye were not dilated; they were not what we call closely contracted; he could not be aroused; the respirations would at times cease, and the heart's action was interrupted or so feeble as to give no pulsation at the wrist; I inquired as to the plan of treatment pursued by young Dr. Shaw; I was told that he had called in the night and found the patient in great distress, and had administered subcutaneously what he regarded as half a grain of morphine, and half an hour later the pain not subsiding he had administered the same quantity in the same way, and had left other powders of morphine of the same style to be administered into the stomach every four hours, if in pain, I think; I learned from the family that none of the powders had been given; I examined and weighed the powders; they contained a little less than one-half a grain of morphine; I inferred if the patient had received no more than two doses of half a grain each he could not be suffering so seriously from the effects; a man under the influence of a grain of morphine so administered would *not die;* he was a thick, short, plethoric, hearty man; did not notice the length of his neck; all men are liable to apoplectic effusions; they said he had eaten a hearty supper; I think the conversation was all in Mrs. Dustin's presence; the act of vomiting might affect the brain, and compression of the brain would produce vomiting; he was comatose; there are various diseases that produce comatose conditions; all men die in one of three ways, by coma, syncope or apnœa; his condition so much resembled apoplexy that I could not discriminate it from that, or from uræmic poisoning; a person who had eaten a hearty supper and gone to bed *upon it* would, perhaps, be more lable to apoplexy; the eyes were not sufficiently contracted to indicate narcotic poison.

Cross-examined.—The pupils were somewhat contracted; in apoplexy the eyes are usually dilated; the administration of one grain of morphine in one-half grain doses within a half hour to a strong man, when in great pain, would not be too much; subcutaneous administration has a greater effect than when taken in the stomach; I do not believe a grain could kill him; never thought Dustin died from an overdose of morphine; I inquired into the circumstances before I came to a conclusion.

Re-examined.—To ascertain how the man died I would have made a post-mortem examination; were it a case of poisoning from opium, in the early part of the case, the patient might be aroused; if morphine is administered subcutaneously the insensibility of the patient will supervene sooner; I think a grain could be administered with safety.

Re-cross examined.—The idiosyncrasy of the patient does have its effect.

Merrill H. Shaw being sworn on the part of the defendant, testified: I am father of the defendant; have known James E. Dustin some years; have formerly treated him; was called to his house between five and six A. M. of the 30th of June last; defendant called for me; found the man in a comatose condition; irregular breathing and irregular pulse; adopted various plans to restore circulation; could not succeed; the pupils of the eye were not much contracted, slightly so; the man had no consciousness; could not be aroused; was there half an hour before Dr. Miner came; applied ammonia to the chest and extremities; my son handed the powders to me; I weighed them. (The defendants offer to prove the weight of the powders—was excluded by the Court.) I remained three-quarters of an honr; there was no change except the gradual sinking of a dying man; I left with Dr. Miner about half past seven; Merrill E. Shaw, the defendant, was a graduate of the Buffalo Medical College in 1864; he served as an assistant surgeon in the 89th and 116th Regiment N. Y. V.; subsequently the defendant pursued the practice of medicine, having his office on Seneca street; Dustin was about five feet seven inches in height; would weigh 160 or 165 pounds; was a person of plethoric habit.

Sylvester F. Mixer sworn for defendant, testified: Have been a practicing physician twenty-six years; such symptoms as are mentioned by Dr. Shaw would be indicated from apoplexy; I have seen persons who died from disorders of the bowels show the same symptoms; I think the treatment by Dr. Shaw was proper; I do not see that the defendant could have done differently; that treatment was not an adequate cause of death; a man who takes a hearty supper and goes to bed on it is more likely to have apoplexy; I have seen patients die from disease of the ileo-cœcal valve with the same symptoms Dustin had; a person would die from uræmic poison from the same symptoms.

Cross-examined.—Morphine produces sickness of the stomach, and will produce dizziness; I have known cases where the eyes were not dilated in apoplexy; could not ascertain what the man died of without a post-mortem examination.

Sandford Eastman sworn for the defendant, testified: I have been a practitioner of medicine and surgery for sixteen or seventeen years; am Professor of Anatomy and Clinical Surgery in Buffalo Medical College; the treatment by Dr. Shaw was perfectly proper; I have given twice the quantity that Dr. Shaw gave without any injury; I frequently give three-fourths of a grain in a dose; a person suffering from pain can take a larger dose than one without pain; if the pain continues the indications would be that the dose was not too large; a person of short stature and short neck would be more liable to apoplexy; the same symptoms might have occurred from half a dozen other causes than morphine; they might have occurred from apoplexy, from inflammation of the stomach, from peritonitis, from disease of the ilio-cæcal valve, and from various other causes; nothing but a post-mortem examination would prove the cause of the death; contraction of the pupils must be extreme to show narcotic poison; a slight contraction would not show it; in apoplexy you can not restore momentary consciousness.

Cross-examined.—In apoplexy the eye is sometimes dilated and sometimes contracted.

Thomas F. Rochester sworn for the defendant, testified: Have been a physician and surgeon for twenty-one years: have been in this city between fourteen and fifteen years; am Professor of the Practice of Medicine in Buffalo Medical College; know the defendant; he graduated at that college; the treatment was proper; ordinarily it would not be an adequate cause of death; the same symptoms are common to poisoning from morphine and other causes; some persons are more quickly affected than others from opium; a person affected with apoplexy would be perfectly unconscious; perforation of the intestines from any cause would produce similar conditions; have seen one instance where such symptoms were produced when the patient was supposed to be narcotized from opium; autopsy showed the cause; in the absence of a post-mortem examination it could not be known from what cause he died; a stout person, of full habit, is more liable to apoplexy; one after eating a hearty supper would be more liable to apoplectic attack; the symptoms described indicate apoplexy.

Cross-examined.—The symptoms described by plaintiff when deceased was sick at first showed that the morphine affected him, but not necessarily that it was an overdose; a small dose will produce dizziness; in the first stage of apoplexy or poisoning from opium the pupils would be contracted, at a later stage they would be dilated.

The defendant rested.

The testimony having closed the counsel for the defendant requested the Court to instruct the jury to render a verdict for the defendant on the ground that whatever might have been the actual cause of death, it appeared indisputably that the treatment resorted to was, under the circumstances, proper and discreet, and that the weight of testimony to that effect was so preponderating that if a contrary verdict should be rendered by the jury it would be the duty of the Court to set it aside.

His Honor, Justice Clinton, said in substance, that the plaintiff had not shown that the morphine which was administered to the deceased had anything to do with his death; that according to the medical testimony the grain of morphine was not an adequate cause of death. The plaintiff having the burden of proof that the death was attributable to the morphine. has wholly failed to establish that proposition. Physicians are often called upon to act in sudden emergencies, in cases of great distress, often when much doubt must necessarily exist as to the remedy. Omniscience and infallibility are not exacted of them. In this case the defendant has acted in a manner which the other physicians who have been sworn have approved as proper treatment of his patient. The defendant therefore stands wholly vindicated and exempt from blame. A verdict was directed for the defendant.

